# Bacterial envelope polysaccharide cues settlement and metamorphosis in the biofouling tubeworm *Hydroides elegans*

**DOI:** 10.1038/s42003-024-06585-9

**Published:** 2024-07-19

**Authors:** Marnie L. Freckelton, Brian T. Nedved, Michael G. Hadfield

**Affiliations:** Kewalo Marine Laboratory, University of Hawaiʻi, Honolulu, HI 96813 USA

**Keywords:** Chemical ecology, Developmental biology

## Abstract

Metamorphosis for many marine invertebrates is triggered by external cues, commonly produced by bacteria. For larvae of *Hydroides elegans*, lipopolysaccharide (LPS) from the biofilm-dwelling bacterium *Cellulophaga lytica* induces metamorphosis. To determine whether bacterial LPS is a common metamorphosis-inducing factor for this species, we compare larval responses to LPS from 3 additional inductive Gram-negative marine biofilm bacteria with commercially available LPS from 3 bacteria not known to induce metamorphosis. LPS from all the inductive bacteria trigger metamorphosis, while LPS from non-inductive isolated marine bacteria do not. We then ask, which part of the LPS is the inductive element, the lipid (Lipid-A) or the polysaccharide (O-antigen), and find it is the latter for all four inductive bacteria. Finally, we examine the LPS subunits from two strains of the same bacterial species, one inductive and the other not, and find the LPS and O-antigen to be inductive from only the inductive bacterial strain.

## Abstract (web summary)

Analysis of products of marine biofilms that induce settlement of invertebrate larvae reveals a specific bacterial LPS component, the O-antigen polysaccharide, to be the inductive cue.

## Introduction

The pivotal role of bacteria and bacterial biofilms in the establishment and maintenance of benthic marine eukaryotic communities is now broadly recognized^1–3^. Bacterial biofilms rapidly coat new surfaces in the ocean, and it is to these bacteria-coated surfaces that small mobile larvae of marine invertebrate animals are recruited. This recruitment process is not random; rather, it is guided by the detection and response to specific surface ‘cues’ that enable larvae to select appropriate habitats, resulting in settlement and metamorphosis^2,3^. However, despite the widespread phenomenon and importance of larval recruitment, understanding the cues and mechanisms underlying these processes remains limited^1–4^.

An equally important role for investigations of bacterial induction of metamorphosis lies in the greater insights it generates into the developmental processes involved^2,5,6^. Metamorphosis for most animals involves massive morphological, physiological, and ecological changes. A swimming, filter-feeding, or non-feeding larva becomes a benthic juvenile, often with a very different food source and mode of feeding^2^. Larva-specific organs are lost, and juvenile-adult structures emerge, typically for feeding and motility. The roles of bacteria in triggering these events demand much greater in-depth investigation, both for the molecular nature of the cues and for the understanding that the cues provide relative to larval receptor systems and how metamorphic signals are disseminated within the larva. Few studies have thus far been definitive in this regard^7–9^ with the exception of the exopolysaccharides that trigger metamorphosis in the hydroid *Hydractinia echinata*^9,10^ and tetrabromopyrrolle that triggers metamorphosis without settlement in Acroporid corals^11^.

An animal that has emerged as an excellent model for the study of bacterial induction of settlement and metamorphosis in marine invertebrates is the serpulid polychaete tubeworm, *Hydroides elegans*^12^. An important and cosmopolitan member of the global warm-water biofouling community, *H. elegans* has been readily adapted to laboratory research due to its fast maturation and ease of propagation^12^. Most importantly, larvae of *H. elegans* will not settle without the presence of a bacterially filmed surface^13^. Furthermore, larvae of *H. elegans* can be induced to settle by some, but not all of the marine biofilm bacterial species that have been investigated. For example, Unabia and Hadfield^14^ found eight of 18 strains of culturable biofilm bacteria-induced settlement and metamorphosis in larvae of *H. elegans*. The inductive bacteria included Gram-positive, Gram-negative, and species with diverse metabolic capacities^14^.

To date, two bacterial cues have been isolated that induce settlement and metamorphosis in *H. elegans*. The first is from the bacterium *Pseudoalteromonas luteoviolacea* HI1, and the second is from the bacterium *Cellulophaga (Cytophaga) lytica*. *P. luteoviolacea* HI1 produces specific complex structures derived from phage-tail proteins, now called tailocins^15^, that are in some manner involved in settlement induction^16–18^. In the case of intact bacterial biofilms, settlement in *H. elegans* is tightly coupled with metamorphosis, with settlement always preceding morphogenesis; that is, larvae attach to the surface and secrete a primary tube before loss of cilia (motility). In contrast, tailocin preparations produce a loss of cilia and the beginnings of morphogenesis before larvae attach to a surface and would be unlikely to produce successful recruitment in a natural setting^5,19^. The research presented here thus focuses on the recent discovery that larvae of *H. elegans* are induced to settle by the outer membrane lipopolysaccharide (LPS) of the Gram-negative, biofilm bacterium *Cellulophaga lytica*^20,21^.

LPS is a highly variable macromolecule located in the outer leaflet of the outer membrane of Gram-negative bacteria^22^ and serves as one of the primary bacterial molecules that animals perceive. LPS can trigger the innate immune response^23^, thereby notifying the animal of potential infection. Conversely, it can also stimulate a positive response, such as in the establishment of the symbiotic relationship between the well-studied Hawaiian bobtail squid *Euprymna scolopes* and its luminous bacterial symbiont, *Vibrio fischeri*^24^. LPS has two major components, lipid A and O-antigen, a polysaccharide. Understanding whether settlement and metamorphosis responses to the LPS from *C. lytica* are induced by the lipid A or O-antigen may reveal important information about how larvae detect this cue, as well as why differences among bacterial strains make them inductive or non-inductive to larvae. Conserved receptors to the lipid-A component of LPS have been identified across the animal kingdom^25^. However, knowing that competent larvae of *H. elegans* must physically contact a biofilm to detect the settlement cue suggests that the outermost, polysaccharide portion of the LPS molecule, the O-antigen, provides that stimulus^13,26^. Making this determination, LPS or O-antigen as the inducer was the first goal of the research described here.

The identification of LPS as an inducer of settlement and metamorphosis in larvae of *H. elegans* also raised the question of whether induction of metamorphosis in this species by LPS is restricted to *C. lytica* or represents a far more widespread inductive element. As noted above, larvae of *H. elegans* respond to a range of bacterial species and strains, potentially reflective of their cosmopolitan habitat. To this end, we examined the LPS from ten Gram-negative bacteria, represented by four species extracted directly from marine biofilms (*Cellulophaga lytica* HI1, *Thalassotalea euphilliae* (two strains: H1 and H2), *Pseudoalteromonas luteoviolacea* (three strains: HI1, B1P, ATCC 33492) and *Tenacibaculum aiptasiae* T48) and three commercially available (*Escherichia coli* O55:B5, *Salmonella enterica* serotype enteritidis and *Pseudomonas aeruginosa* ATCC 27316 serotype 10). Finally, we broke the LPS macromolecules from each of the inductive bacteria into their lipid and polysaccharide components and identified which component of LPS is responsible for the inductive action.

## Results

### O-antigen is the inductive component of LPS isolated from *C. lytica* HI1

**Fig. 1:**
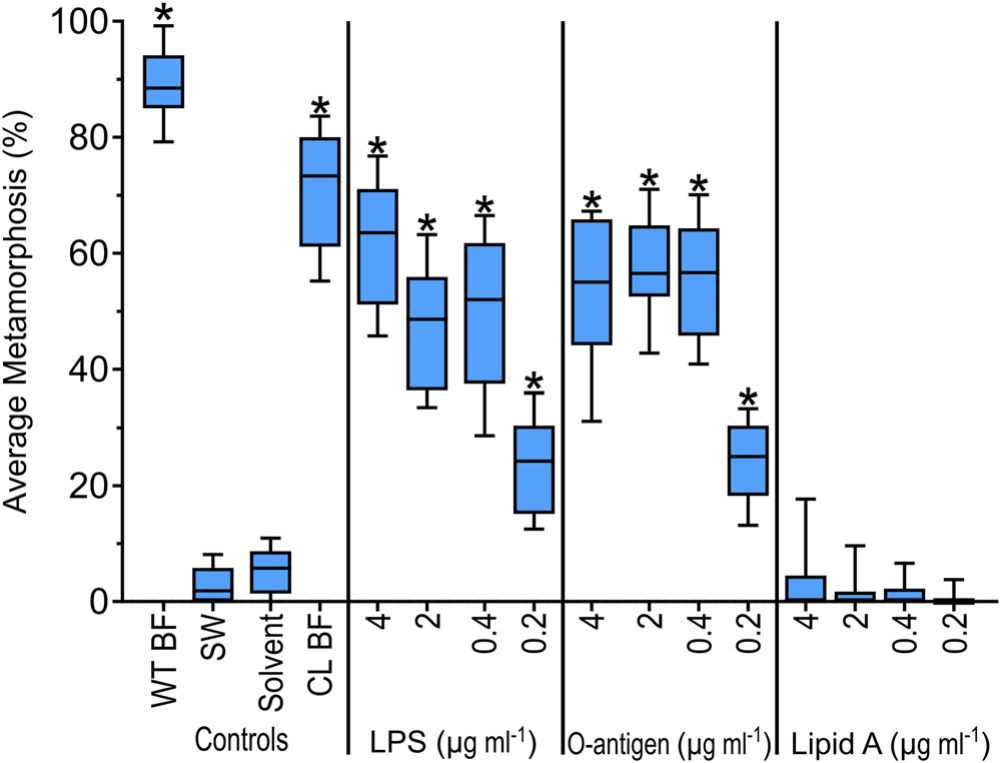
Larvae of *Hydroides elegans* settle and metamorphose in response to O-antigen from the bacterium *Cellulophaga lytica* HI1. Settlement and metamorphic responses from larvae of *Hydroides elegans* after 24 h exposure to the Lipopolysaccharide (LPS), O-antigen, and Lipid A extracted from the bacterium *C. lytica* HI1 are depicted as a boxplot. The central horizontal line represents the median, the lower and upper boundaries of the box indicate the 25th and 75th quartiles, and the whiskers extend to the 10th to 90th percentiles; *n* = 12. Asterisks show significant differences from SW (*p* < 0.05). Note: a methanol solvent control was included for lipid A samples; methanol was evaporated overnight before the addition of larvae. WTBF wildtype biofilm, SW sterile seawater, CL BF a *C. lytica* biofilm inoculated at 10^8^ cells ml^−1^.

A yield of 0.5386 g of LPS was isolated from 5 g of freeze-dried cells of *C. lytica* HI1. SDS-PAGE and TLC confirmed the purity and identity of the isolated LPS (Supplementary Fig. 1). Mild acid hydrolysis of 500 mg of this LPS yielded 280 mg of O-antigen and 190 mg of lipid A after lyophilization. Significant settlement and metamorphosis were observed for the biofilm, LPS, and O-antigen samples (*H*_(15,12)_ = 171.7; *p* < 0.05) (Fig. 1). No significant settlement (or metamorphosis) was observed for the lipid A samples (Fig. 1). The highest induction of metamorphosis for both the LPS and O-antigen samples from *C. lytica* HI1 was obtained using a concentration of 4 µg ml^−1^ (Fig. 1).

### Active LPS and O*-*antigen from other inductive Gram-negative marine bacteria induce settlement and metamorphosis in larvae of *H. elegans*

**Fig. 2:**
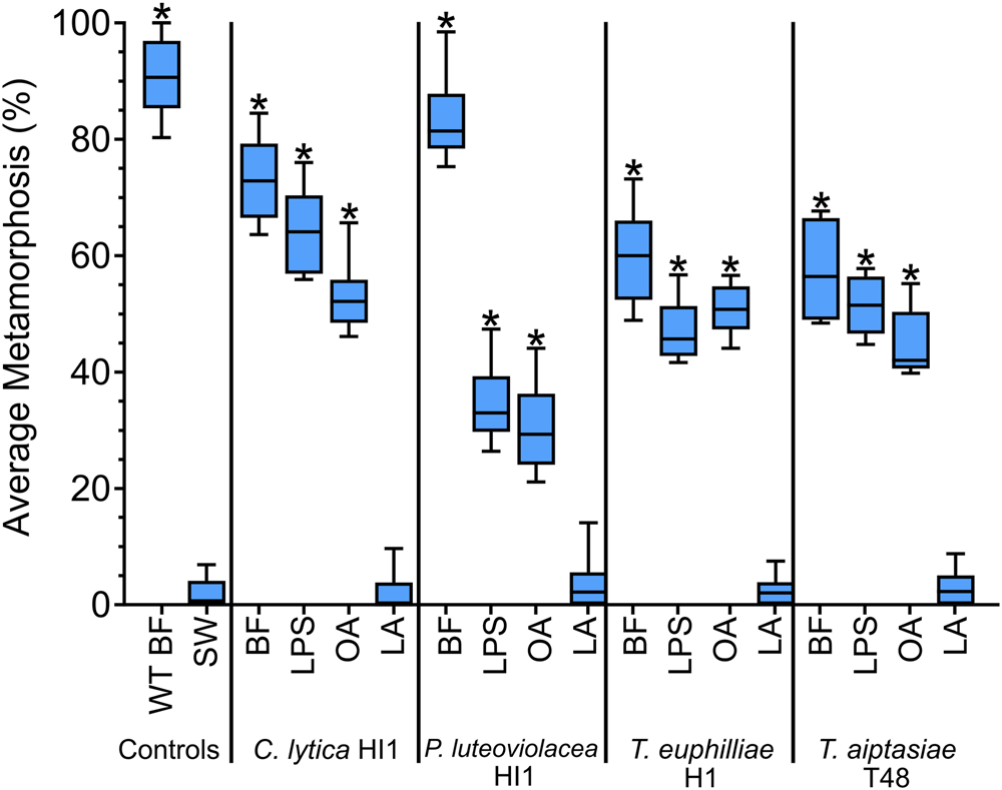
Larvae of *Hydroides elegans* settle and metamorphose in response to O-antigen from marine bacteria *Pseudoalteromonas luteoviolacea* HI1, *Thalassotalea euphilliae* H1, and *Tenacibaculum aiptasiae* T48. Settlement and metamorphic responses from larvae of *Hydroides elegans* after 24 h exposure to monospecific biofilms, extracted lipopolysaccharide (LPS), O-antigen (OA), and lipid A (LA) *C. lytica* HI1, *P. luteoviolacea* HI1, *T. euphilliae* H1, and *T. aiptasiae* T48 are depicted as a boxplot. The central horizontal line represents the median, the lower and upper boundaries of the box indicate the 25th and 75th quartiles, and the whiskers extend to the 10th to 90th percentiles; *n* = 12. Monospecific biofilms were inoculated at 10^8^ cells ml^−1^, except *P. luteoviolacea* HI1 biofilm which was inoculated at 10^7^ cells ml^−1^. Extracts were tested at 4 µg ml^−1^. Asterisks show significant differences from SW (*p* < 0.05). WTBF wildtype biofilm, SW sterile seawater, BF biofilm.

Larvae of *H. elegans* were induced to settle and metamorphose by monospecific biofilms, LPS, and isolated O-antigens from *Cellulophaga lytica* HI1, *Pseudoalteromonas luteoviolacea* H1, *Thalassotalea euphilliae* H1, and *Tenacibaculum aiptasiae* T48 (*H*_(15,12)_ = 200.0; *p* < 0.05; Fig. 2). However, Lipid A samples from these strains did not induce metamorphosis (Fig. 2).

### Commercial LPS and O-antigen samples do not induce metamorphosis in larvae of *H. elegans*

**Fig. 3:**
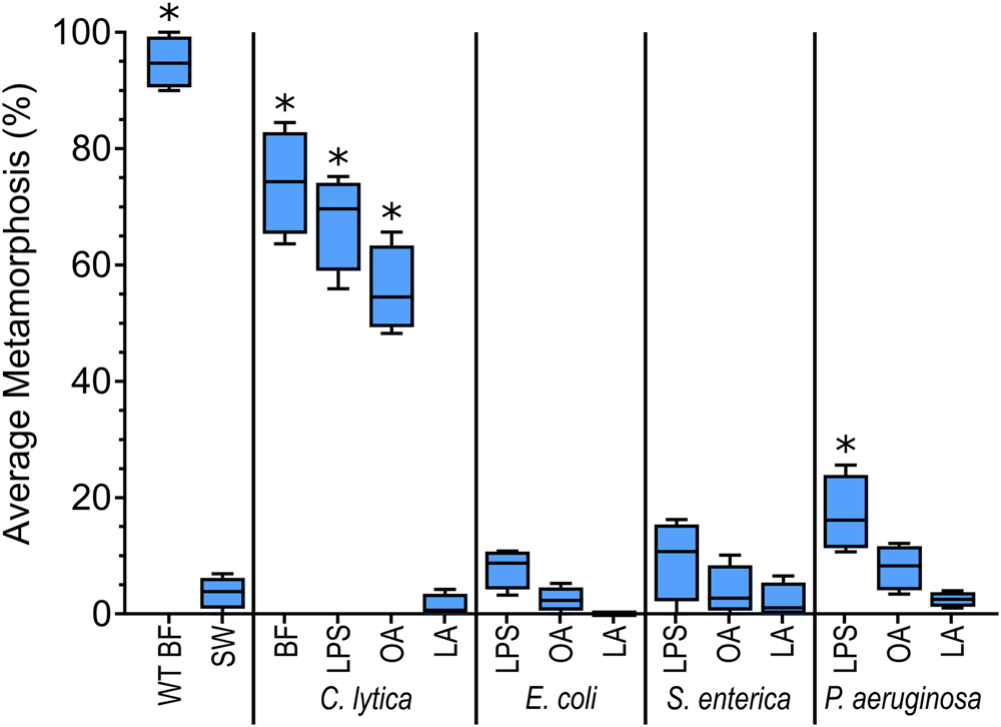
Larvae of *Hydroides elegans* do not settle and metamorphose in response to commercial LPS or O-antigen from *Escherichia coli* O55:B5, *Salmonella enterica* serotype enteritidis and *Pseudomonas aeruginosa* ATCC 27316. Boxplot depicting the Settlement and metamorphic responses from larvae of *Hydroides elegans* after 24 h exposure to lipopolysaccharides (LPS), O-antigen (OA), and lipid A (LA) from *Cellulophaga lytica* HI1, *E. coli* O55:B5, *S. enterica* serotype enteritidis and *P. aeruginosa* ATCC 27316 tested at 4 µg ml^−1^. *C. lytica* biofilms were included for reference and inoculated at 10^8^ cells ml^−1^. The central horizontal line represents the median, the lower and upper boundaries of the box indicate the 25th and 75th quartiles, and the whiskers extend to the 10th to 90th percentiles; *n* = 12. Asterisks show significant differences from DFASW (*p* < 0.05).

Of the commercial samples, only the LPS from *P. aeruginosa* ATCC 27316 serotype 10 induced settlement and metamorphosis that was significantly greater than the negative control, (*H*_(14,12)_ = 146.4; *p* < 0.05; Fig. 3), while significantly less than both the positive control and the reference samples from *C. lytica* (Fig. 3). Neither LPS nor O-antigen from *E. coli* O55:B5 and *S. enterica* serotype enteritidis induced settlement or metamorphosis in larvae of *H. elegans* (Fig. 3).

### Different bacterial strains have contrasting effects on the induction of settlement and metamorphosis

**Fig. 4:**
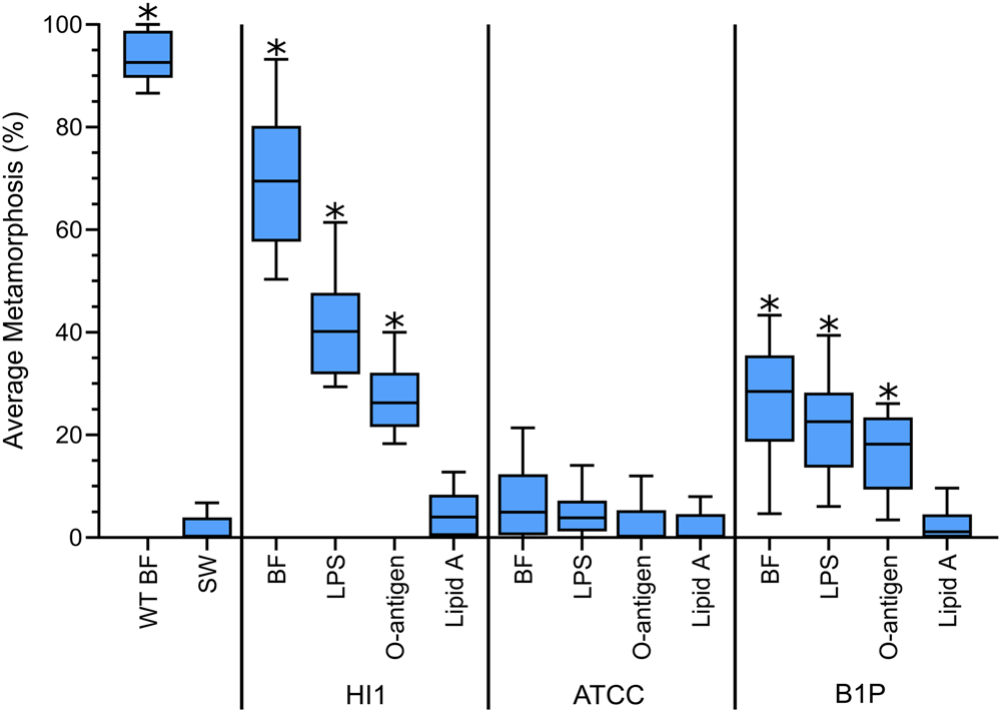
Larvae of *Hydroides elegans* settle and metamorphose in response to O-antigen from inductive strains of *Pseudoalteromonas luteoviolacea.* Boxplot depicting settlement and metamorphosis responses in larvae of *Hydroides elegans* after 24 h exposure to isolated lipopolysaccharide (LPS), O-antigen (OA), and lipid A (LA) from three strains of *Pseudoalteromonas luteoviolacea* (HI1, ATCC 33492, B1P). Extracts were tested at 4 µg ml^−1^. The central horizontal line represents the median, the lower and upper boundaries of the box indicate the 25th and 75th quartiles, and the whiskers extend to the 10th to 90th percentiles; *n* = 12. Asterisks show significant differences from SW (*p* < 0.05). Note: a methanol solvent control was included for lipid A samples; methanol was evaporated overnight before the addition of larvae. WTBF wildtype biofilm, SW sterile seawater.

**Fig. 5:**
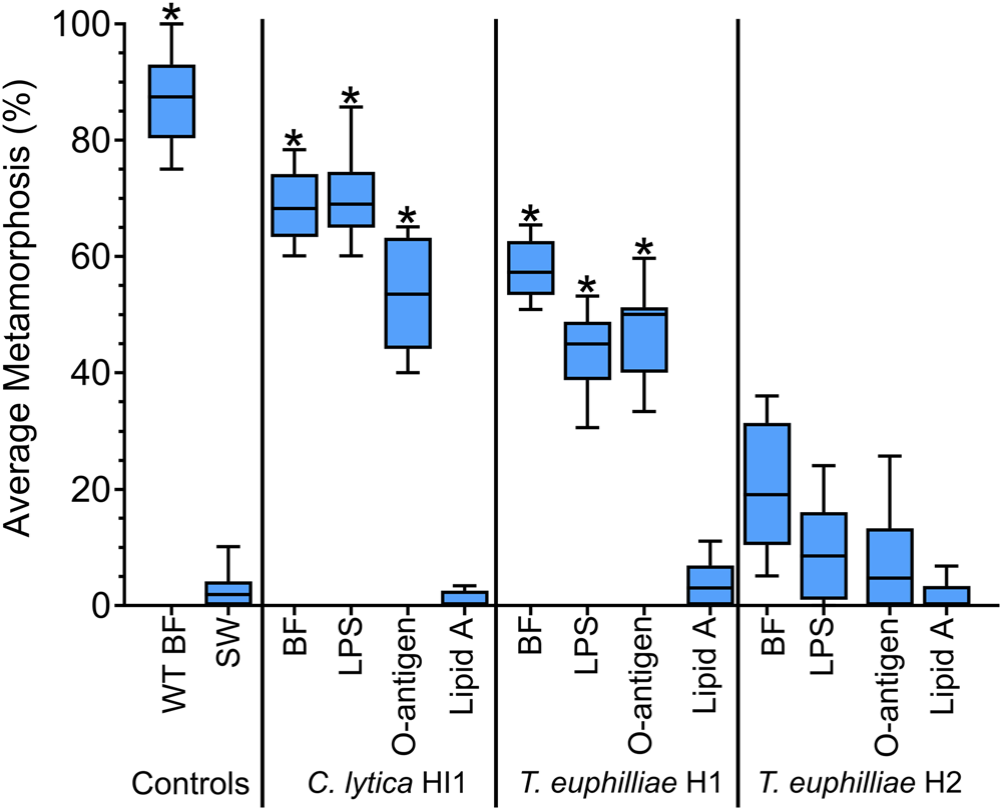
Larvae of *Hydroides elegans* settle and metamorphose in response to O-antigen from the inductive strain of *Thalassotalea euphilliae.* Boxplot depicting settlement and metamorphosis in larvae of *Hydroides elegans* after 24 h exposure to isolated lipopolysaccharide (LPS), O-antigen (OA), and lipid A (LA) from two strains of *T. euphilliae* (H1 and H2). Extracts were tested at 4 µg ml^-1^. The central horizontal line represents the median, the lower and upper boundaries of the box indicate the 25th and 75th quartiles, and the whiskers extend to the 10th to 90th percentiles; *n* = 12. Asterisks show significant differences from SW (*p* < 0.05). A methanol solvent control was included for Lipid A samples. Note: a methanol solvent control was included for lipid A samples; methanol was evaporated overnight before the addition of larvae. WTBF wildtype biofilm, SW sterile seawater.

Larvae of *H. elegans* were strongly induced by the biofilm from *P. luteoviolacea* HI1 and moderately by strain B1P, but not at all by strain ATCC 33492 (*H*_(13,12)_ = 139.3; *p* < 0.05; Fig. 4). Larvae of *H. elegans* were moderately induced by the LPS and O-antigen isolated from both *P. luteoviolacea* HI1 and B1P but not at all by strain ATCC 33492 (Fig. 4). Larvae of *H. elegans* were strongly induced by the biofilm, LPS, and O-antigen from *T. euphilliae* H1, not at all by strain *T. euphilliae* H2. (*H*_(13,12)_ = 151.0; *p* < 0.05; Fig. 5). No lipid A samples were inductive (Figs. 4 and 5).

## Discussion

Our criteria for accepting a cue as ecologically relevant require it to be bacterial in origin, fast-acting, and biofilm-associated. The discovery that O-antigen from *Cellulophaga lytica* induces settlement and metamorphosis in *Hydroides elegans* larvae advances our understanding of the induction process, complementing our previous findings that lipopolysaccharide (LPS) from the same bacterium is inductive. This consistency in inducing capabilities further clarifies the role of these molecules in mediating larval settlement and metamorphosis and explains why the larvae must physically contact a biofilm for induction to occur. O-antigen, as the outermost component of LPS, is structurally positioned for this essential contact.

In almost all settlement assays, the monospecific biofilms provided the highest incidence of settlement and metamorphic induction. The drop in observable metamorphosis when larvae were exposed to LPS and O-antigen was rarely significant, with the exception of *P. luteoviolacea*, and most likely reflects the transition from surface-bound cues in the biofilm assays to the bath exposures of the assays with extracted components. The requirement for surface-bound cues for the natural induction of settlement and metamorphosis *H. elegans* is well-established^13^. Our new understanding of the role of LPS and more specifically the O-antigen component further supports this requirement; glycolipids and polysaccharides frequently possess more than a single low-energy conformation^27–29^. The steric environment of the membrane is likely to favor a particular conformation, but this pressure is removed upon extraction of the molecule^27–29^. The complex nature of the biofilm may also contribute additional inductive components, but previous comprehensive experiments on the bacterium *Cellulophaga lytica*^21^ suggest that this is unlikely. Further, the experimental results presented here suggest that O-antigen is also the primary inductive component for *T. euphilliae* and *T. aiptasiae* but not for *P. luteoviolacea*, for which an additional inductive cue has already been identified^16^.

Given the prevalence of LPS and O-antigen in Gram-negative bacteria, we next asked, whether other Gram-negative bacteria that induce settlement and metamorphosis in larvae of *H. elegans* also did so through LPS and O-antigen. The involvement of LPS in immune responses and inter-organismal interactions is well-established. LPS is a potent microbe-associated molecular pattern (MAMP)^25^. MAMPs are conserved molecules or molecular motifs produced by microorganisms (both pathogenic and beneficial) that are recognized by the innate immune systems of animals and plants^25^. LPS, found on the outer membrane of Gram-negative bacteria serves as a key identifier of both potential pathogens^30^ and potential beneficial symbionts^31,32^. Given this widespread involvement in immune responses and inter-organismal interactions, it is unsurprising that our study confirmed that the pattern of inductive LPS and O-antigen extended across all four of the isolated marine bacterial biofilm species. Conserved molecules and detection systems involved in the interactions between eukaryotes and bacteria enable faster determination of friend from foe, or, in the case of marine larvae, “good” habitats from “bad.”

Interestingly, although LPS is a prime example of a MAMP, most pathogenic interactions that occur through this pathway are focused on the lipid A or endotoxin portion of the molecule and not the O-antigen polysaccharide. However, the role of carbohydrates in the stimulation of metamorphosis in marine invertebrates is far from new^33–35^, with increasing numbers of papers appearing that implicate a role for bacterial polysaccharides in the induction of a wide range of marine invertebrates^26,33^. Importantly, strong evidence for conserved motifs within metamorphic inductive cues may also point to conserved receptors for settlement and metamorphic cues across invertebrate phyla.

The bacterium *Pseudoalteromonas luteoviolacea* strain HI1, a highly inductive biofilm species, isolated and genetically characterized in the Hadfield lab^18,36,37^ presents a challenge to interpret. Recently, a closer examination of the bacterial cues produced by *P. luteoviolacea* provides important insight and criteria for assessing the potential for inductive cues to explain bacterial induction of metamorphosis on a broad scale. While the inductive potential of LPS isolated from *P. luteoviolacea* was significantly lower than that of the bacterium in a monospecific biofilm or of the tailocin complex^5,16^, it was significantly greater than sterile water (Fig. 4). This is consistent with previous studies that showed a loss of activity with the filtration of tailocin preparations^16,20^. One explanation for this could be that larvae of *H. elegans* respond so strongly to biofilms of *P. luteoviolacea* HI1 because multiple modes of action are in play. Multiple modes of action would be consistent with the settlement behavior in larvae of *H. elegans;* they are early colonizers of biofilms in the field, and respond to multiple taxonomically varied bacteria, resulting in their widespread distribution. Additionally, a recent detailed examination of the order of settlement and metamorphic events in larvae of *H. elegans* revealed that metamorphosis induced by tailocin extracts does not follow the same order of events as that of wild biofilms or biofilms of *P. luteoviolacea* HI1 alone and would not be likely to produce successful attachment in a natural setting^5,19^. It should be stressed that this does not mean that *H. elegans* is a poor model organism, but rather imposes a filter of ecological relevance upon any identified inductive cues.

It should be noted that although *P. luteoviolacea* and *C. lytica* are readily isolated and cultured, both appear to be rare in marine biofilms^38^, including those from a harbor where *H. elegans* is abundant^38,39^. This mismatch of culture-dependent and culture-independent studies raises the question, are marine larvae induced to settle based on the presence of rare bacteria, or do many bacteria, easily cultured and not, can produce inductive cues? For this reason, the strain *Tenacibaculum aiptasiae* T48, isolated from biofilms collected in Pearl Harbor, Hawaiʻi, was included in our study. The same investigation that revealed *Pseudoalteromonas* and *Cellulophaga* to be rare genera in biofilms showed *Tenacibaculum* to be strongly represented in inductive bacterial biofilms^38^. This indicates that for larvae of *H. elegans* both rare and abundant bacterial strains induce metamorphosis and suggests that the structure of the LPS polysaccharide is more important than the abundance of one specific bacterial producer. The appearance of similar inductive cues in both rare and abundant bacteria also establishes the possibility of synergistic or additive effects on the inductive capacity of wild biofilms and may explain why wild biofilms are almost always more inductive than monospecific biofilms. That is, larvae of *H. elegans* are not solely dependent on the presence of a single, rare but highly inductive bacterium such as *P. luteoviolacea* HI1 but rather can respond to whichever bacteria produce the correct motif for induction. This concept fits with both the prevalence of LPS as well as the prevalence of *H. elegans* worldwide and will be important to explore as this field progresses.

The discovery of contrasting bioactivity among different strains of the same bacterial species, particularly in their ability to induce settlement and metamorphosis in *H. elegans* larvae, highlights a significant but often overlooked aspect of bacterial ecology: strain-level differences. These differences are crucial, however, as they can profoundly impact the functional properties of bacteria and their interactions with other organisms. In pathogenic studies, it is well known that some bacterial species, e.g., *Vibrio cholerae*, can produce both pathogenic and non-pathogenic strains^40,41^. However, strain-level differences are often overlooked in microbiome research, potentially leading to oversimplified conclusions about how bacteria and eukaryotes interact. This new understanding suggests that the variation in the chemical structure of LPS molecules, influenced by species, strains, and environmental conditions, is one way that marine biofilms provide valuable information about the suitability of a site for the settlement and metamorphosis of marine invertebrate larvae.

The close linkage between environments, bacterial biofilms, and metamorphosis induction in marine invertebrates is increasingly apparent^42,43^. However, as climate change becomes our current reality, there are many questions to which we still have very few answers. The responses of biofilm members to environmental conditions may result in biofilms with different species compositions and biochemical fingerprints such that they can represent a varied source of information on the suitability of a site for the settlement and metamorphosis of specific marine invertebrate larvae. The secondary impacts of marine biofilms stimulating the recruitment of macro-organisms to surfaces are both positive and negative^1,2^. Induction of settlement and metamorphosis in the larvae of food species (e.g., clams, oysters, and mussels), or habitat-forming organisms (e.g., corals) is a highly desirable outcome^2^. However, the recruitment of larvae to the bottoms of ships and industrial equipment in the sea^44^, known as biofouling, constitutes a strong negative outcome. To date, most investigations of marine microbial communities have focused on either host-associated microbiomes or planktonic microbes largely with the focus of avoiding disease in existing populations^45–49^. In contrast, very few studies focus on the coming climate impacts on the biofilms that establish and maintain these communities. As climate change continues to threaten the health, resilience, and persistence of marine benthic communities^50^, we must better understand the role of bacterial biofilms in stimulating settlement and metamorphosis in marine invertebrates and the processes and drivers that establish and maintain healthy and robust benthic marine communities. The interplay between bacteria-induced development and the ecological impact of the interaction is clear, emphasizing anew the importance of investigating development and ecology together^51^.

## Methods

### Larval culture

Metamorphically competent larvae of *Hydroides elegans* were produced as previously described^12^. Briefly, dioecious adult *H. elegans were* collected from Pearl Harbor (Hawaiʻi USA) and maintained in continuously flowing, unfiltered seawater at the University of Hawaiʻi’s Kewalo Marine Laboratory. Adult worms were induced to spawn by gentle removal from their tubes, and gametes were mixed. Larvae were fed the single-celled alga *Isochrysis galbana* Tahitian Strain at a concentration of approximately 60,000 cells ml^−1^. The metamorphically competent nectochaete stage was reached after five days of culture and was utilized in metamorphosis experiments for 1–2 days at ~26 °C.

### Bacterial culture

*Cellulophaga lytica* HI1^37,52^, *Pseudoalteromonas luteoviolacea* strains: H1^36,37^, ATCC 33492^T^^53^, B1P^54^; *Thalassotalea euphilliae* strains: H1^54,55^, H2^54,56^, and *Tenacibaculum aiptasiae* T48, all Gram-negative bacteria isolated from biofilms in Hawaiʻi (Supplementary Table 1), were streaked from −80 °C glycerol stocks onto half seawater tryptone agar (1/2FSWt)^57^ and incubated at 25 °C for 24–48 h. Single colonies were used to inoculate 3 ml broth cultures and incubated for 4 h at 28 °C with shaking (170 rpm). These starter cultures were adjusted to an OD_600_ of 1.000 and aliquots were used to inoculate overnight cultures. Cells were harvested by centrifugation (4000 *g*, 30 min, 4 °C), and washed with 1/10th volume of double-filtered autoclaved seawater (DFASW). Bacterial cells were then either frozen for later LPS extraction or diluted for biofilm formation.

### Monospecific biofilms

Washed bacterial cells were resuspended in DFASW with volume adjusted to produce a cell density of 10^8^ cells ml^−1^ for all strains except *P. luteoviolacea* as previously described^16,21,37^. Briefly, adjusted bacterial suspensions were added to 24-well plates and incubated for 1 h at room temperature ( ~ 26 °C) to enable the attachment of cells. Biofilmed wells were gently washed three times with DFASW to remove unattached cells. Biofilms were then ready for settlement and metamorphosis assays. Biofilm formation and density were confirmed for each bacterial strain (at the time of assessment) by staining additional replicates with 0.1% crystal violet solution for 10 min^58^ (Supplementary Fig. 1). Stained biofilms were gently washed to remove excess stain and then dried. The crystal violet was then solubilized in ethanol and the absorbance was read at 590 nm.

### LPS extraction and purification

Lipopolysaccharides (LPS) were extracted from all bacteria strains using Apicella’s modification of the hot phenol method^59,60^ as previously described^21^. LPS extracts were purified by adding 50% aqueous trichloroacetic acid (CCl_3_CO_2_H) at 4 °C to precipitate proteins and nucleic acids. The precipitate was removed via centrifugation (1100 *g*, 4 °C, 30 min), after which the supernatant was dialyzed against distilled water and lyophilized to provide the corresponding LPSs. All LPS samples were checked for contaminating proteins, nucleic acids, and other lipid classes using spectroscopy, TLC, and ProQ Emerald 300 (Invitrogen) as previously described^21^ before being used in settlement and metamorphosis assays or further chemical isolations. Each LPS fraction was assessed for metamorphic induction in larvae of *H. elegans* at 4 µg ml^−1^ and 8 µg ml^−1^, this reflects the optimal amount of LPS within a monospecific biofilm of *C. lytica* as previously determined^21^.

### O-antigen: Lipid A separation

O-antigens were separated from lipid A components of LPS through mild acid hydrolysis with 2% aqueous acetic acid at 100 °C until lipid precipitation (6 h). The precipitate was removed by centrifugation (13,000 x *g*, 20 min)^61^, and the supernatant was collected and lyophilized in preparation for settlement and metamorphosis assays. Samples were spectrographically checked for the presence of contaminating lipids (O-antigen samples) or polysaccharides (lipid A samples) and found to be free of lipid or polysaccharides, respectively.

### Commercially available LPS samples

LPS extracted from non-marine, human pathogenic strains (*Escherichia coli* O55:B5, *Salmonella enterica* serotype enteritidis, and *Pseudomonas aeruginosa* ATCC 27316 serotype 10) is commercially available (Sigma Aldrich). Samples were purchased from each to establish whether LPS needed to be produced by a marine strain of bacteria isolated from a wild bacterium. Commercial LPS samples were treated the same as for the extracted LPS, above, and subjected to the same purification steps as the extracted samples. Commercial samples were purchased, because the bacteria were not available for use, and, as such, no biofilm treatments were carried out for these species.

### Settlement and metamorphosis assays

Settlement and metamorphosis assays were carried out in 24-well plates, with 20–50 larvae per well and four replicates per treatment from 3 separate spawning events from different individuals. The percent of larvae that metamorphosed was determined at 24 h. Because larvae of *H. elegans* will not metamorphose without exposure to bacterial biofilms or their products, natural, complex films developed on glass slides suspended for approximately one month in the laboratory’s unfiltered seawater supply served as positive larval controls^38,62,63^. Double-filtered, autoclaved seawater (DFASW) served as a negative larval control. For experiments that contained lipid A, which is not water-soluble, samples were first dissolved in methanol, transferred to experimental wells, and then evaporated before the addition of larvae; to control for this methanol solvent controls were included where the equivalent amount of methanol was added and evaporated to ensure that any activity was due to the lipid A and not to any residual methanol. Settlement and metamorphosis assays also included a biofilm of *C. lytica* for reference.

### Statistics and reproducibility

All statistical analyses were performed in GraphPad Prism 9 for Windows, GraphPad Software, San Diego, California USA, www.graphpad.com. Significant differences (*p* < 0.05) were calculated using Kruskal-Wallis followed by Dunn’s test for multiple comparisons with false detection rate (FDR) correction^64^.

### Supplementary information

Supplementary Material

## Data Availability

All raw data^65^ that supports this study has been deposited in the Figshare repository and can be found at 10.6084/m9/figshare.24030090.
