# Supplementary material for: Bacterial envelope polysaccharide cues settlement and metamorphosis in the biofouling tubeworm Hydroides elegans
Source: Commun Biol. 2024 Jul 19;7:883. doi: 10.1038/s42003-024-06585-9 (PMC11271524; doi:10.1038/s42003-024-06585-9)
Supplement: Supplementary file 1 — Supplementary Material [file 42003_2024_6585_MOESM1_ESM.docx]

Supplementary Material for “Bacterial envelope polysaccharide cues settlement and metamorphosis in the biofouling tubeworm *Hydroides elegans*.”

Marnie L Freckelton^1^, Brian T Nedved^1^ and Michael G Hadfield^1^*

Affiliations: ^1^ Kewalo Marine Laboratory, University of Hawaiʻi, Honolulu, Hawaiʻi, United States, 96813.

*Author for correspondence

Corresponding author’s email address: hadfield@hawaii.edu

Supplementary Table 1. Bacterial Isolates used in this study

| **Species** | **Strain** | **Accession #** | **Isolated from** | **Inductive/ Non-Inductive** |
| --- | --- | --- | --- | --- |
| *Cellulophaga lytica* | HI1 | CP009239^1,2^ | Pearl Harbor | Inductive |
| *Pseudoalteromonas luteoviolacea* | HI1 | JWIC00000000^3^ | Pearl Harbor | Inductive |
|  | ATCC 33492 | GCA_001625655^4^ | Sediment | Non-Inductive |
|  | B1P | HQ439502^5^ | Kaneohe Bay | Non-Inductive |
| *Thalassotalea euphilliae* | H1 | HQ439526^5^ | Kaneohe Bay | Inductive |
|  | H2 | HQ439504^5^ | Kaneohe Bay | Non-Inductive |
| *Tenacibaculum aiptasiae* | T48 | PP473977 | Pearl Harbor | Inductive |

Supplementary Figure 1. Crystal violet staining of monospecific biofilms of each bacterial isolate tested. Biofilms were inoculated at 10^8^ cells/ml except for *P. luteoviolacea* stains which were inoculated at 10^7^ cells/ml. Absorbance was read at 590 nm.

**References:**

1. Huang, S. & Hadfield, M. G. Composition and density of bacterial biofilms determine larval settlement of the polychaete *Hydroides elegans*. *Mar. Ecol. Prog. Ser*. **260**, 161–172 (2003).
2. Asahina, A. Y. & Hadfield, M. G. Complete genome sequence of *Cellulophaga lytica* HI1 using PacBio single-molecule real-time sequencing. *Genome Announc*. **2**, e01148–14 (2014).
3. Asahina, A. Y. & Hadfield, M. G. Draft genome sequence of *Pseudoalteromonas luteoviolacea* HI1, determined using Roche 454 and PacBio single-molecule real-time hybrid sequencing. *Genome Announc*. **3**, e01590–14 (2015).
4. Gauthier, G., Gauthier, M., & Christen, R. Phylogenetic analysis of the genera *Alteromonas*, *Shewanella*, and *Moritella* using genes coding for small-subunit rRNA sequences and division of the genus *Alteromonas* into two genera, *Alteromonas* (Emended) and *Pseudoalteromonas* gen. nov., and proposal of twelve new species combinations. *Int. J. Syst. Evol. Microbiol.* **45**, 755-761 (1995).
5. Tran, C. & Hadfield, M. G. Larvae of *Pocillopora damicornis* (Anthozoa) settle and metamorphose in response to surface-biofilm bacteria. *Mar. Ecol. Prog. Ser.* **433**, 85–96 (2011).
